# Jejunal Gastrointestinal Stromal Tumor: A Rare, Elusive, and Formidable Cause of Obscure Bleeding

**DOI:** 10.7759/cureus.71286

**Published:** 2024-10-11

**Authors:** Vincent Wong, Ravi Upadhyay, Umair Nasir, David Friedel

**Affiliations:** 1 Gastroenterology, NYU (New York University) Langone Health, Mineola, USA; 2 Internal Medicine-Pediatrics, Rutgers University New Jersey Medical School, Newark, USA

**Keywords:** bleeding gist, gastrointestinal endoscopy, gastrointestinal stromal tumor (gist), obscure gastrointestinal bleeding, recurrent gi bleeding

## Abstract

Gastrointestinal bleeding is classified as obscure in 5% of patients who remain symptomatic after undergoing upper endoscopy, colonoscopy, and small bowel capsule endoscopy. We present a case of a 45-year-old male who had obscure bleeding for eight years and presented with hemorrhagic shock. He was found to have an ulcerated intra-luminal jejunal lesion on enteroscopy, then had surgical resection that revealed a low-risk gastrointestinal stromal tumor (GIST). If GISTs are bleeding, hemostasis should first be achieved with medical, endoscopic, or radiologic interventions, and then resected because they can have malignancy potential. Furthermore, they should be risk-stratified and either surveilled to monitor for recurrence if low risk or need adjuvant imatinib if high risk. There are currently no screening guidelines for GISTs despite their increasing incidences.

## Introduction

Gastrointestinal (GI) bleeding can have a mortality rate of 5-10%, so timely diagnosis and interventions are essential [1]. The most common causes of GI bleeding in the upper GI tract seen on endoscopy are peptic ulcer disease (40% of patients) and Mallory-Weiss tears (15%) [1-3]. In the lower GI tract, the most common causes of GI bleeding seen on colonoscopy are diverticular bleeding (65%) or angiodysplasias (15%) [1-3]. In the small bowel, 30% of bleeds can be from angiodysplasias, which may be visualized on video capsule endoscopy or enteroscopy [1-3]. Rare causes include aortic-enteric fistulas, anastomotic ulcers, benign or malignant tumors, and Dieulafoy lesions [1-3]. In about 5% of cases, bleeding is classified as obscure when no identifiable source is found [3]. We present a report of a 45-year-old male with obscure GI bleeding for eight years despite multiple endoscopies who was found to have a gastrointestinal stromal tumor (GIST) in the jejunum.

## Case presentation

A 45-year-old male with a history of hypertension, gastroesophageal reflux disease, and multiple episodes of obscure GI bleeding for the past eight years presented with lightheadedness and melena for one day. He denied abdominal pain, nausea, vomiting, or hematochezia. He did not take non-steroidal anti-inflammatory drugs (NSAIDs) and was not on anticoagulation or antiplatelet medications.

The GI procedures performed over the last eight years are listed in Table 1.

**Table 1:** Gastrointestinal procedures performed for the patient prior to admission

Time since index presentation	Type of procedure	Findings
Index	Esophagogastroduodenoscopy (EGD)	Multiple fundic gland polyps without bleeding
	Colonoscopy	Within normal limits
	Video capsule endoscopy	Streaks of blood seen at 19% into the small bowel
	Anterograde double balloon enteroscopy	Within normal limits
1 year	Retrograde double balloon enteroscopy	Within normal limits
2 years	Enteroscopy	Multiple gastric polyps without bleeding
	Video capsule endoscopy	Within normal limits
26 months	EGD	Multiple gastric polyps without bleeding
3.5 years	EGD	Multiple gastric polyps without bleeding
	Enteroscopy	Within normal limits
6.5 years	EGD	Multiple non-bleeding gastric polyps without bleeding
7 years	Video Capsule Endoscopy	Healing erosion seen at 37% into small bowel without active bleeding

Upon arrival, the patient was hypotensive to 89/48 mmHg and tachycardic to 102 beats per minute. Notable laboratory values are listed in Table 2.

**Table 2:** Laboratory values for the patient on arrival

Type of laboratory study	Patient’s lab value	Reference values
White blood cell count (10^3/uL)	13.3	4.2-9.1
Hemoglobin (g/dL)	7.9 (baseline of 12-15)	13.7-17.5
Mean corpuscular volume (fL)	79.1	79-92
Red blood cell distribution width (%)	48.3	11.6-14.4
Blood urea nitrogen (mg/dL)	34 (baseline of 10)	8-26
Creatinine (mg/dL)	1.2	0.7-1.3
Prothrombin time (sec)	11.7	9.9-13.1
International normalized ratio	1.02	0.86-1.14
Partial thromboplastin time (sec)	25.9	25.7-37.6

**Figure 1:**
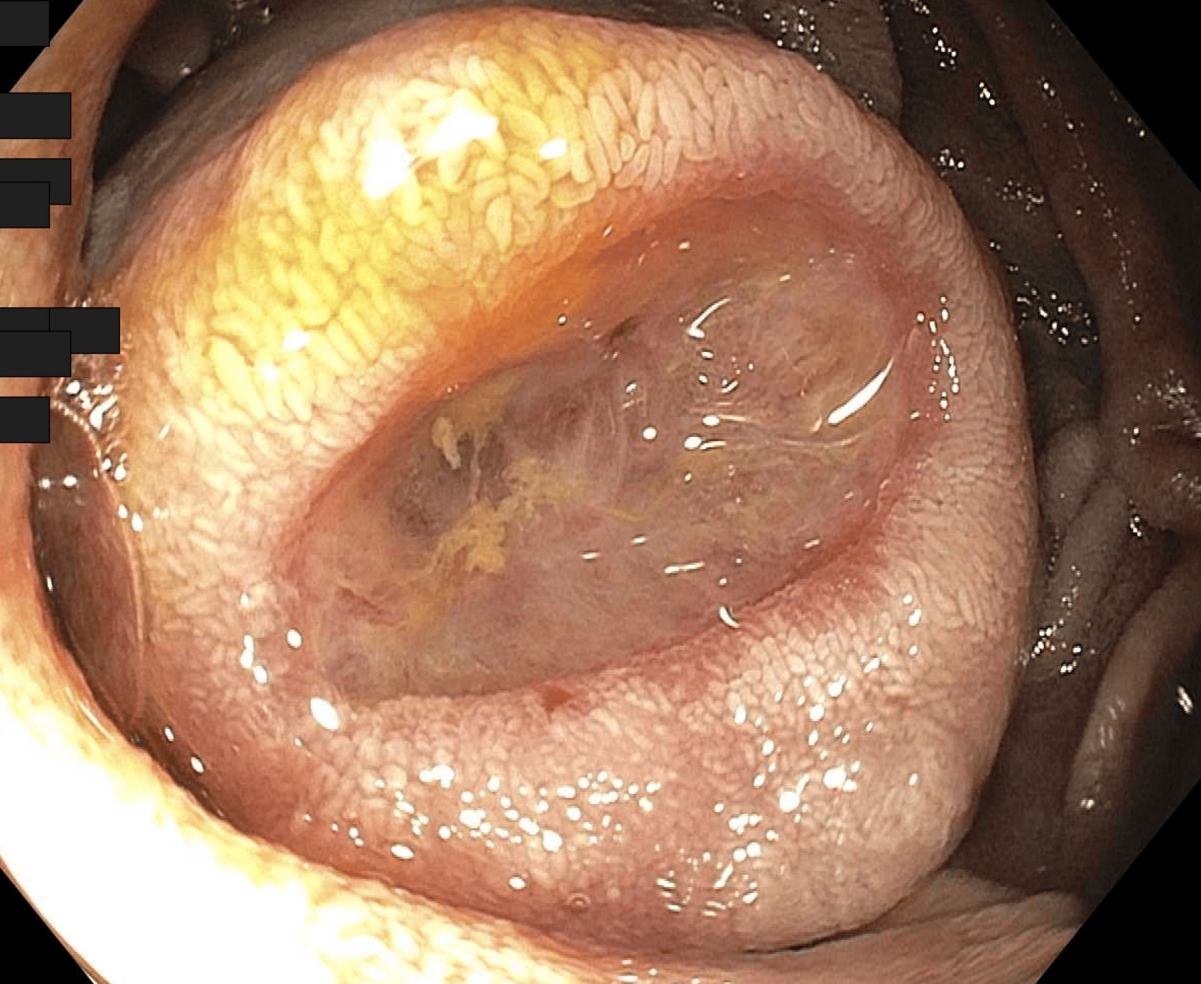
Luminal view of ulcerated jejunal polyp with cherry red spot diagnosed as gastrointestinal stromal tumor

He was given intravenous fluids and started on intravenous pantoprazole. After medical optimization, he had an enteroscopy that revealed a sessile polyp at the jejunum with stigmata of recent bleeding (Figure 1).

**Figure 2:**
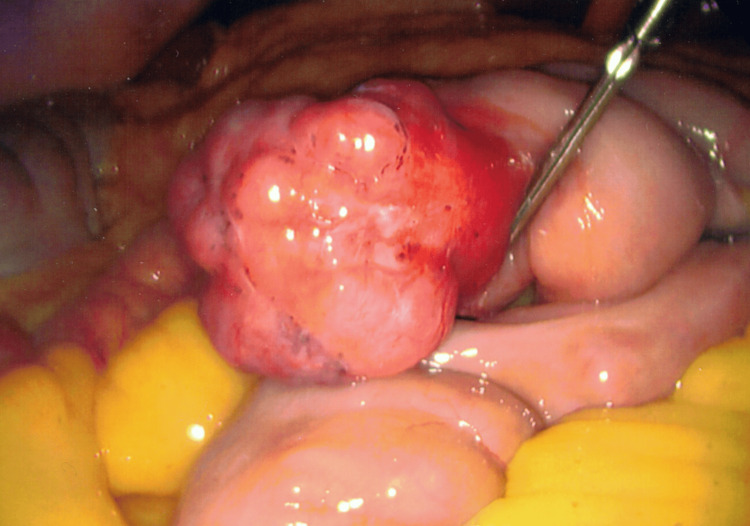
Serosal view of jejunal gastrointestinal stromal tumor

The area adjacent to the lesion was marked with India ink and a hemostatic clip. He subsequently had an exploratory laparotomy where the lesion was located and resected (Figure 2).

Pathologic examination showed a 3.5-centimeter pT2N0 low-risk gastrointestinal stromal tumor that was positive for the c-KIT mutation in exon 11 but negative for the platelet-derived growth factor receptor alpha (PDGFRA) mutation. Positron emission tomography (PET) scan did not show signs of metastasis.

After resection of the mass, he did not have further episodes of GI bleeding and was discharged from the hospital. Because the mass was greater than 2 centimeters and localized, he was advised to have a computed tomography (CT) scan or magnetic resonance imaging (MRI) scan of the abdomen every three to six months for the next three to five years to monitor for recurrence. However, he was lost to outpatient follow-up.

## Discussion

GISTs are hypervascular mesenchymal stromal tumors originating from the interstitial cells of Cajal and comprising 0.1-3% of all GI malignancies [4,5]. They are usually found incidentally in patients with a mean age of 60 in the stomach (60%) and the small bowel (30%) [4,5]. They can be asymptomatic (20%), bleed if ulcerated (30%), or cause intestinal obstruction (30%) when they grow intraluminally [4,5]. Their malignancy potential is based on size, location, mitotic index, genetics, and composition [4,5]. They can invade the liver, peritoneum, and greater omentum, unlike other sarcomas [4,5]. The diagnosis is made endoscopically and often incidentally. Visualization may be difficult as these lesions can be intramural, extraluminal, or mimic other pathologic lesions [4]. Patients may need multiple endoscopies as GISTs can be subtle and cause occult GI blood loss [1-3].

Bleeding GIST lesions located in the stomach can be seen on upper endoscopy [4-7]. However, enteroscopy, colonoscopy, and video capsule endoscopy may be needed to identify more distal lesions. Once the bleeding lesion is found, clips, endoloops, argon plasma coagulation, thermal coagulation, direct injections of epinephrine, and hemostatic sprays can be applied for temporary hemostasis [6-9]. Interventional radiology can also inject hemostatic agents into vessels that feed the tumor; however, this comes with risks for pancreatitis, intestinal ischemia, arterial dissection, off-target embolization, and hematoma at the access site [8]. There has been a case report mentioning the use of octreotide in the management of GIST tumor bleeding [10]. Octreotide causes rapid, prolonged vasoconstriction of splanchnic vessels, lowering gastric mucosal blood flow and inhibiting acid and pepsin secretion to help with clot stabilization [10]. 

The definitive therapy for bleeding lesions is resection of the lesions [4,7]. They can be removed endoscopically if they are small but they are usually surgically excised [4,7,11]. R0 resection rate is not as high as that of traditional surgery, with the risk for GI tract perforation, tumor bleeding, and rupture [4]. R1 resection should not warrant repeat surgery [4]. Bleeding GISTs indicate increased risks for infection, perforation, and tumor recurrence [12]. High-risk tumors are treated with adjuvant imatinib and followed by CT, MRI, or PET scans every three to six months although surveillance intervals may vary based on the literature [4,5,11]. Low-risk tumors may be followed with imaging every six to twelve months for five years postoperatively [4,5,11]. There is no documented role for endoscopic surveillance after resection [11].

There are currently no screening modalities for GIST tumors despite their increasing incidences in the last few decades [13]. If there is suspicion of subepithelial lesions, endoscopic ultrasound can be performed to characterize and biopsy them [11,14]. Next generation sequencing techniques such as circulating tumor DNA and miRNAs can also be used to help diagnose and stratify GIST tumors [11]. Lesions that are less than two centimeters and do not have any high-risk features may be monitored endoscopically once every year [11,14]. If these small tumors have high-risk features or are greater than or equal to two centimeters, surgery is the preferred treatment [11,14]. There are no surveillance guidelines for patients who do not undergo surgery [11].

Our patient underwent multiple upper endoscopies, enteroscopies, video capsule endoscopies, and a colonoscopy over eight years before the source of bleeding was identified. The GIST lesion was found to be intra-luminal and ulcerated with stigmata of recent bleeding. No endoscopic interventions were performed as it was not actively bleeding, but the adjacent area was marked with India Ink and a clip since surgical resection was anticipated. Despite the patient’s clinical presentation and endoscopic findings, the tumor was positive for the C-kit mutation, localized, and had a low mitotic rate, so it was considered to be low risk [11,15]. Imaging surveillance was recommended but he was lost to follow-up [11,15].

## Conclusions

GISTs located in the jejunum can be difficult to identify. They can cause obscure gastrointestinal bleeding and may lead to an extensive workup with multiple endoscopic procedures. When found, these lesions should be resected, risk-stratified, treated with imatinib if necessary, and monitored for recurrence. There are currently no screening guidelines for GIST lesions, but their increasing incidences should prompt further research to identify lesions before they cause severe bleeding.
